# Supplementary material for: Cancer awareness among adolescents in Irish schools: A cross-sectional study
Source: PLoS One. 2025 Mar 12;20(3):e0319252. doi: 10.1371/journal.pone.0319252 (PMC11902254; doi:10.1371/journal.pone.0319252)
Supplement: S2 File — (DOCX) [file pone.0319252.s002.docx]

Supplementary file 2

**Length of time before seeking medical help for a symptom you thought might be cancer**

|  | **Overall (n=474)** | |  | **Gender (n=466)^a^** | | | | | |  | **Knowing someone with cancer (n=455)^b^** | | | | | |
| --- | --- | --- | --- | --- | --- | --- | --- | --- | --- | --- | --- | --- | --- | --- | --- | --- |
|  |  |  |  | **Male (n=224)** | |  | **Female (n=242)** | |  |  | **Yes (n=421)** | |  | **No (n=34)** | |  |
|  | **%** | **(n)** |  | **%** | **(n)** |  | **%** | **(n)** | **p-value^c^** |  | **%** | **(n)** |  | **%** | **(n)** | **p-value^c^** |
| Within 24 hours | 28.3 | (134) |  | 27.2 | (61) |  | 29.3 | (71) | **0.011** |  | 29.0 | (122) |  | 17.6 | (6) | 0.515 |
| Between 2 to 3 days | 24.5 | (116) |  | 28.6 | (64) |  | 20.2 | (49) |  |  | 24.2 | (102) |  | 26.5 | (9) |  |
| Between 4 and 10 days | 19.4 | (92) |  | 21.4 | (48) |  | 17.4 | (42) |  |  | 18.8 | (79) |  | 17.6 | (6) |  |
| Within a month | 14.3 | (68) |  | 10.7 | (24) |  | 18.2 | (44) |  |  | 14.0 | (59) |  | 23.5 | (8) |  |
| Longer than a month | 4.0 | (19) |  | 1.8 | (4) |  | 6.2 | (15) |  |  | 4.0 | (17) |  | 5.9 | (2) |  |
| Don't know/Did not answer | 9.5 | (45) |  | 10.3 | (23) |  | 8.7 | (21) |  |  | 10.0 | (42) |  | 8.8 | (3) |  |

^a^data on gender not available for n=3 and gender=Other for n=5. Hence, n=8 excluded from analysis.

^b^data on knowing someone with cancer not available for n=19. "Knowing someone with cancer" is defined as the participant having had cancer themselves or knowing a close family member, a friend or someone else that had cancer.

^c^from Fisher's exact test comparing all 6 categories.
